# Data on the relationship of signal-to-cutoff ratio of two HIV antigen/antibody combination assays to subsequent confirmation of HIV-1 infection in a low-prevalence population

**DOI:** 10.1016/j.dib.2020.105707

**Published:** 2020-05-16

**Authors:** Christina K. Hodgson, Matthew D. Krasowski, Bradley A. Ford

**Affiliations:** Department of Pathology, University of Iowa Hospitals and Clinics, 200 Hawkins Drive, Iowa City, IA 52242, USA

**Keywords:** False positive reaction, HIV-1, HIV-2, immunoassay, polymerase chain reaction, viral load

## Abstract

HIV-1/2 antigen/antibody (Ag/Ab) immunoassays that detect HIV-1 and HIV-2 antibodies and HIV-1 p24 antigen are commonly used in the diagnosis of HIV-1/HIV-2 infections in human plasma/serum. Samples from patients with positive screening results require confirmation by antibody differentiation and/or HIV PCR assays. HIV screening assays are commonly reported as positive or negative based on a signal-to-cutoff (S/CO) threshold. For some HIV screening assays, the strength of the S/CO value correlates with likelihood that confirmatory testing will be positive. The data in this article provide results from two HIV Ag/Ab combination assays (Abbott Architect HIV Ag/Ab Combo Assay, a 4^th^ generation combination assay; Bio-Rad Bioplex 2200 HIV Ag-Ab Assay, a 5^th^ generation assay). The data include 23,331 HIV screening results, S/CO ratios, antibody differentiation or Western blot results (for samples with positive HIV screens), HIV-1 PCR results (if performed), patient location at time of testing, age, and sex. Distribution of S/CO ratios for the Bio-Rad HIV screening assay data and the distribution of S/CO values for samples with positive screening results were analyzed.

Specifications Table

**Table:** 

Subject	Medicine and Dentistry
Specific subject area	Pathology and Medical Technology
Type of data	Tables Figures Supplemental files
How data were acquired	Retrospective chart and data review from laboratory analysis performed at an academic medical center central clinical laboratory were obtained via tools within the electronic medical record.
Data format	Raw and Analyzed
Parameters for data were collection	Retrospective data on all HIV screening and confirmatory tests was obtained from the electronic medical record (Epic, Inc.) covering the time period from January 1, 2014 through May 24, 2017. Detailed chart review was performed for all cases with positive HIV screening results except for testing for blood borne pathogen (BBP) exposures that were restricted from chart review. The project had approval from the University of Iowa Institutional Review Board.
Description of data collection	There were a total of 23,331 HIV screening tests performed on 19,177 (Architect 9,302, Bioplex 9,875) unique patients for clinical purposes during the retrospective analysis period. The study was a retrospective study approved by the University of Iowa Institutional Review Board (protocol # 201705802). The data collection also contains confirmatory HIV testing for positive screens.
Data source location	Iowa City, Iowa, United States of America
Data accessibility	Raw data are available in this article as 2 Supplementary files. Five tables and two figures are included within the paper.

## Value of the Data

• The data provided are of value as HIV screening is widely performed in clinical, research, and public health settings.
• Clinicians, other researchers, or personnel in clinical laboratories might find this data useful as a reference for comparison.
• Our data set would serve as a starting point for researchers interested in future investigations investigating HIV screening false positives.
• The data are of value as there is very limited published data involving the relationship of signal/cutoff ratio and confirmation in 5^th^ generation HIV screening tests.
• The data provide information for 23,331 HIV screening tests in 19,177 unique patients.

## 1 Data

In this retrospective analysis study, we compiled detailed data on 23,331 samples originating from 19,177 unique patients that had HIV screening testing performed at an academic medical center central clinical laboratory. There is a growing literature on the association of HIV screening assay signal-to-cutoff (S/CO) value with likelihood of subsequent confirmation by confirmatory testing such as Western blot, antibody differentiation assays, and HIV RNA PCR assay [1], [2], [3], [4], [5], [6]. There were a total of 2,657 (Architect 2,002; 655 Bioplex) tests ordered as part of workup for BBP exposure to employees or students. For the purposes of the present study, a true (confirmed) positive was determined by a positive result by Western blot, Bio-Rad Multispot, or Bio-Rad Geenius (note only Multispot and Geenius assays can differentiate between HIV-1 and HIV-2 Ab) and/or HIV RNA PCR. A non-true positive screen was defined as a reactive HIV screen without subsequent confirmation/diagnosis of HIV infection. To best ascertain HIV status, we performed extensive chart review in addition to analyzing the results of HIV discriminatory and RNA PCR testing.

**Fig. 1:**
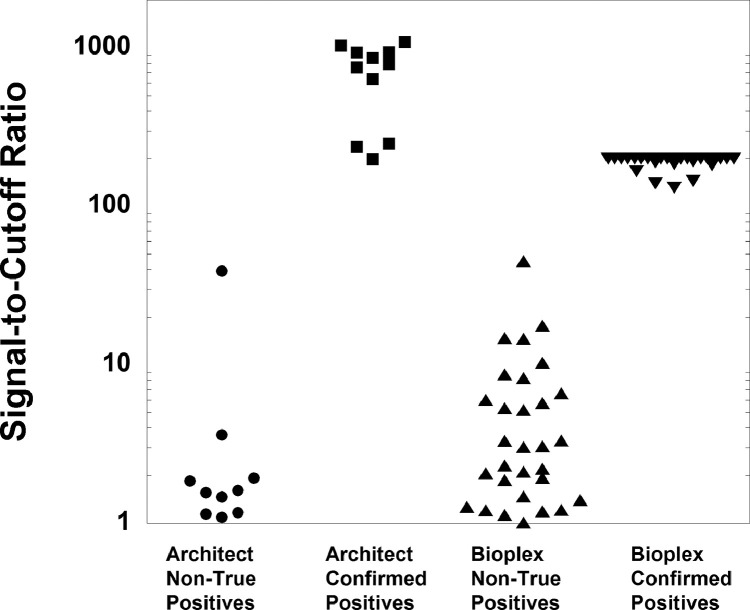
Distribution of S/CO ratios for Confirmed Positives and Non-True Positive Results. Note: 19 Bioplex specimens had S/CO ratios that exceeded 200 (plotted together at 200).

**Fig. 2:**
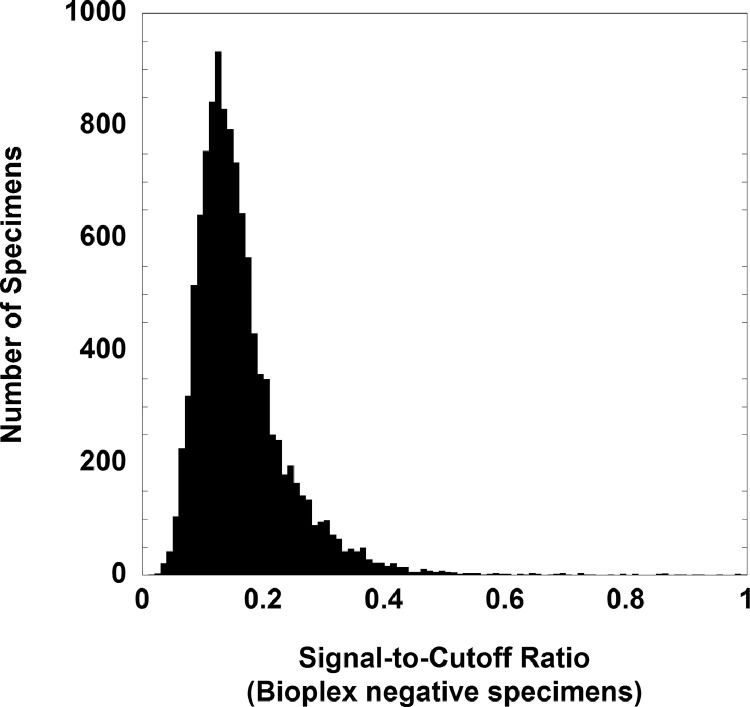
Distribution of S/CO ratios of negative Bioplex screens.

Table 1 shows demographics of the population being tested, both overall and for those with positive screening tests. Table 2 shows overall performance characteristics of the two HIV screening assays. Table 3 shows the S/CO quantitative values associated with reactive HIV screens. Table 4 summarizes the discrete components for reactive HIV screens for the 5th generation Bioplex assay. Table 5 summarizes the performance of the two HIV screening tests using selected S/CO ratio thresholds. Fig. 1 displays the distribution of S/CO ratios for positive HIV screens for the Abbott Architect and Bioplex assays, indicating no overlap between the S/CO ratios for non-true positives and confirmed positives for either assay. Fig. 2 is a histogram plot of the S/CO ratios of HIV screens that were negative (< 1.0) on the Bioplex assays. The raw data for the study are included in Supplementary file 1 (Abbott Architect assay) and Supplementary file 2 (Bioplex assay).

• Supplementary file 1: Data for 11,987 HIV screening tests on 9,302 unique patients using the Abbott Architect 4^th^ generation HIV assay. All laboratory data involve analysis on serum/plasma. Specific data fields include: unique patient identification number (deidentified), location/unit at time of testing (emergency department, inpatient, or outpatient), age in years, birth sex, HIV screening result, S/CO ratio (if available), confirmation method (Multi-spot or Western blot, if performed), confirmation result (if available), and HIV-1 PCR results (if available).
• Supplementary file 2: Data for 11,344 HIV screening tests on 9,875 unique patients using the Bioplex 5^th^ generation HIV assay. All laboratory data involve analysis on serum/plasma. Specific data fields include: unique patient identification number (deidentified), location/unit at time of testing (emergency department, inpatient, or outpatient), age in years, birth sex, HIV screening result, S/CO ratio (if available), confirmation method (Multi-spot or Geenius blot, if performed), confirmation result (if available), and HIV-1 PCR results (if available).

**Table 1:** Demographics of HIV testing

	Architect	Bioplex
HIV screening	n = 11987	n = 11344
**Location**		
Inpatient	1517 (12.7%)	1259 (11.1%)
Outpatient	10218 (85.2%)	9790 (86.3%)
Emergency department	252 (2.1%)	295 (2.6%)
**Highest ordering specialty**		
Obstetrics-gynecology	2567 (21.4%)	3555 (31.3%)
Family medicine	1352 (11.3%)	1372 (12.1%)
Transplant services	867 (7.2%)	772 (6.8%)
Internal medicine	539 (4.5%)	558 (4.9%)
**Confirmed positive screen**		
Mean age (yrs)	46 ± 11	41 ± 13
Median age (yrs)	46	40
Male: female	3:1	3:1
**Non-true positive screen (initial screen positive, not confirmed)**		
Mean age (yrs)	40 ± 15	39 ± 14
Median age (yrs)	33	34
Male: female	0.6	0.8:1

**Table 2:** Performance of Architect and Bioplex HIV Ag/Ab assays

	Architect	Bioplex	Total
**HIV screening**	n = 11987	n = 11344	n = 23331
Reactive screens	52	58	110
Nonreactive screens	11935	11286	23221
**HIV confirmation**			
Confirmed positives	22	27	49
Positive Western Blot	7	N/A	7
HIV-1 by Multispot	14	18	32
HIV-2 by Multispot^a^	1	0	1
HIV-1 by Geenius	N/A	9	9
Non-true positives (confirmation negative)	26	31	57
Confirmatory test cancelled^b^	4	0	4†
**Specificity**			
(95% CI)	99.8% (99.7 - 99.9%)	99.7% (99.6 - 99.8%)	99.8% (99.7 - 99.8)%
**Positive Predictive Value**			
(95% CI)	45.8% (36.6 -55.4%)	46.6% (38 -55.3%)	46.2% (39.9 -52.7%)

a HIV-2 RNA was detected (but below the limited of quantification, <10 copies/mL) by subsequent HIV-2 PCR performed at the University of Washington (Seattle, WA) and New York State Department of Health Wadsworth Center (Albany, NY.) This sample was also screen positive on the Bioplex assay when tested as part of validation studies.

b Confirmatory testing was cancelled in 4 cases for the Abbott Architect testing due to previously established HIV-1 diagnosis.

**Table 3:** S/CO Ratio Quantitative Values for Reactive Screens

Architect HIV Ag/Ab Combo Assay^a^		
S/CO ratio	Negative confirmation	Positive confirmation
	n = 11	n = 12
Median	2	826
Minimum	1	199
Maximum	39	1094

Bioplex HIV Ag/Ab Combo Assay^b^		
S/CO ratio	Negative confirmation	Positive confirmation
	n = 31	n = 27
Median	3	>200
Minimum	1	131
Maximum	45	>200 (n = 19)

a S/CO ratios were only available for 23 of 52 Architect positive screens. A single patient with a reactive Architect result had confirmed HIV-2 infection. Initial HIV testing was performed in this patient as part of routine screening for solid organ transplant evaluation. The quantitative S/CO ratio on the Architect assay was 237.48. The sample was later tested as part of validation studies for the Bioplex assay and had an S/CO ratio of > 200 for HIV-2 Ab (<1 for the two HIV-1 discrete components). HIV-2 antibodies were detected by Multispot confirmation. HIV-2 RNA was detected although below the assay's limit of quantitation (<10 copies/mL) by real-time PCR (University of Washington Medical Center, Seattle, WA). HIV-2 RNA remained detectable at 8 and 9 months by real-time PCR but was no longer detected at 19 months from initial screening.

b S/CO ratios had maximum upper limit of 200 for the Bioplex. All values above this were simply reported as > 200.

**Table 4:** Discrete component for reactive Bioplex screens

Reactive component (S/CO ratio ≥ 1)	HIV-1 Ab	HIV-2 Ab	HIV-1 p24 Ag	HIV-1 Ab, HIV-2 Ab^a^	HIV-2 Ab, HIV-1 p24 Ag^a^	HIV-1 Ab, HIV-2 Ab, HIV-1 p24 Ag^a^
Confirmed positive screens (n = 27)	27	0	0	0	0	0
Non-true positive screens (n = 31)	22	1	0	2	1	5

a These Bioplex patterns combined cross-reactivity for HIV-1 Ab and HIV-2 Ab and are designated in the assay package insert as “undifferentiated HIV”, a result pattern uncommonly associated with actual HIV infection.

**Table 5:** Performance with Selected S/CO Ratio Thresholds

Abbott Architect HIV Ag/Ab Combo Assay		
S/CO ratio^a^	PPV (%)	Sensitivity (%)
>200	100%	100%
>150	100%	100%
>100	100%	100%

Bio-Rad Bioplex HIV Ag/Ab Combo Assay		
S/CO ratio	PPV (%)	Sensitivity (%)
>200	100%	70.4%
>150	100%	88.9%
>100	100%	100%

a S/CO quantitative values were available for 23 of 52 reactive Architect screens and 58 of 58 reactive Bioplex screens.

## 2 Experimental Design, Materials, and Methods

This was a retrospective study approved by the University of Iowa Institutional Review Board (protocol # 201705802). During the retrospective analysis period, initial HIV testing was performed using the Abbott Architect HIV Ag/Ab immunoassay (Abbott Laboratories, Architect Park, IL) January 1, 2014 to November 22, 2015, which then transitioned to the Bio-Rad Bioplex 2200 HIV Ag-Ab Assay (Bioplex Laboratories, Hercules, CA) November 23, 2015 to May 24, 2017. The Abbott Diagnostics Architect HIV Ag/Ab Combo Assay is a widely used chemiluminescent 4^th^ generation assay that simultaneously detects HIV-1 p24 Ag, HIV-1 gp41 Ab, and HIV-2 gp36 Ab and outputs a single signal-to-cutoff (S/CO) ratio that translates into a reactive/nonreactive qualitative result [5,[7], [8], [9]. An S/CO ratio of 1.0 or greater on the Architect assay is considered positive, with repeat testing to verify the result. The Bio-Rad Bioplex 2200 HIV Ag-Ab Assay is an automated, multiplex flow 5^th^ generation HIV immunoassay that provides discrete S/CO ratio results from detection of HIV-1 p24 Ag, HIV-1 Ab (groups M and O), and HIV-2 Ab, with similar overall sensitivity and specificity to other combination HIV Ag-Ab assays [10,11]. An S/CO ratio of 1 or greater on any of the three components of the Bioplex assay is interpreted as a positive screen, with repeat testing for all Bioplex positive screens before proceeding to confirmatory testing [12].

Beginning March 2015, quantitative S/CO ratios were captured in the laboratory information system and were thus available for 23 of 52 reactive Architect screens and all Bioplex HIV screens. The S/CO ratio for the Bioplex assay was determined by the highest S/CO ratio of the three components (HIV-1 p24 Ag, HIV-1 Ab groups M and O, and HIV-2 Ab). Reflex discriminatory or antibody differentiation testing for reactive screens (S/CO ratios of 1.0 or greater) was performed using Western blot for HIV-1 referred to a commercial reference laboratory (ARUP Laboratories, Salt Lake City, UT) January 2014 to October 2014, Bio-Rad Multispot HIV-1/HIV-2 Antibody Differentiation (Bio-Rad Laboratories, Redmond, WA) October 2014 to December 2016, or Bio-Rad Geenius HIV-1/HIV-2 Antibody Differentiation (Bio-Rad Laboratories, Redmond, WA) December 2016 to May 2017. These discriminatory tests, if positive, can confirm HIV infection. Medical record review was performed for all cases with S/CO ratio ≥ 1, except for cases in which HIV testing was ordered on a source patient, exposed employee, or student as part of a blood-borne pathogen (BBP) exposure workup (e.g., needlestick or scalpel injury). Due to restricted access, data for HIV screening in BBP exposures was limited to HIV screening and confirmation laboratory results.

## Appendix Supplementary materials

Image, application 1

Image, application 2

Image, application 3
